# Natural Compounds as Antimicrobial Agents

**DOI:** 10.3390/antibiotics9050217

**Published:** 2020-04-29

**Authors:** Carlos Manuel Franco, Beatriz I. Vázquez

**Affiliations:** Hygiene, Inspection and Food Control Laboratory, Analytical Chemistry, Nutrition and Bromatology Department, Faculty of Veterinary, University of Santiago de Compostela, 27002 Lugo, Spain

## Abstract

During the first two decades of this century, conventional antimicrobial compounds have been found out to have more bacterial resistance. What has also been worrying is the rediscovery of the so-called “natural compounds”, which in turn have a good name among the average citizen because of the former’s plant or animal origin. However, they do not form a well-classified group of substances. This Special Issue consists of five reviews focusing on clinical bacteria applications in food and their specific effects upon virulent bacterial factors. You will also find a research on much needed, new antimicrobials sourced in extreme environments, and secondary metabolites of *Burkholderia*. This issue includes 12 original research papers which will provide you with an in-depth coverage of the protein extract activity, as well as the activity of other plant extracts, on fighting bacteria, fungi or diarrhea. Their use in broilers or laying eggs for production purposes has also been focused on in order to improve gut microbiota. Last but not least, we should not forget about honey and its effect; Allium sativum-fermented extracts, as well as other “natural” compounds, have been studied in their fight against biofilms. Furthermore, we have also examined the use of essential oils, which are currently used in edibles such as fresh sausages. The present work also deals with other applications such as natural compound derivatives as well as compound mixtures.

This book details the manuscripts in the Special Issue of Antibiotics: “Natural Compounds as antimicrobial agents”. Our in-depth study comprises 17 manuscripts, which focus on an important group of aspects related to biocontrol once this wide range of compounds has been used. Firstly, we start with two interesting reviews; one which deals with the use of traditional medicinal plants against clinical pathogens, documenting [1] a huge series of minimal inhibitory concentrations, using over 200 medicinal plants to fight pathogens off. The other is an analysis of the major applications of natural compounds for one of their main uses—food preservation so as to enhance food safety—in a review by prof. Quinto and colleagues [2]. Edible films as well as nanoparticles are also included. The food use of these compounds is perceived benevolently by consumers, so not only was the study of natural compounds as growth bacteria inhibitors examined in this present issue, but also the effect of these compounds on the virulent related factors of specific bacteria, i.e., T3SS (Type III Secretion System used by many Gram negative bacteria [3]). Further in the issue, you will also find two interesting reviews on the conventional meaning of “Natural Compounds” and conventional antibiotics, between which the borders are very thin. So thin, in fact, that many believe in the richness of extreme ecosystems for finding Actinobacteria-producing antibiotics [4], and in the use of secondary metabolites from *Burkholderia* as new sources in antibiotic development [5].

Additionally, there are, as has been stated above, 12 research papers studying, firstly [6], the effect of protein extracts from *Loranthus europaeus* berries against phytopathogenic fungi as well as foodborne bacteria. The already mentioned extracted experiments have shown an important activity against bacteria, though no effect against fungi was found. Bear in mind that you will also find the use of medicinal plants in this use against diarrhea [7]. This highlights an interesting aspect that needs further study as far as the metal content of these plants is concerned. Another research paper studies the properties of witch-hazel plants and Green tea extracts upon the pathogenesis inhibition of staphylococci [8]. In another extract worth mentioning, Hibiscus activity against multidrug-resistant bacteria is thoroughly described [9], along with the elucidation of the extract compounds by means of magnetic resonance spectroscopy (as well as other techniques). Two more papers covering the research on the use of natural compounds in animal production are also included within this Special Issue. They comprise broiler and laying hens, using Chicory (*Chicorium intybus*) [10] resulting in gut microbiota improving. Fermented defatted Alperujo is also focused upon, including its role in enhancing the absorption capacity of intestinal mucosa [11]. Furthermore, not only have we studied the effect of natural compounds on gut microbiota, but we have also covered those natural compounds produced by gut microbiota—the short-chain fatty acids (studied by Lamas et al. [12]; the latter study regarding the effect on the biofilm formation, gen expression, and motility of *Salmonella*). Their effect against biofilm has also been observed using both honey [13] and *Allium sativum* fermented extract, and cannabinol oil extract [14]. The essential oil of *Zataria multiflora* and hops extracts have also been tested in fresh sausages [15], so as to avoid using other conventional preservatives. They have shown, however, that using natural compounds does not always imply an antimicrobial effect, which introduces the need to study which types of application are most advisable for these compounds. Finally, two extra chapters focusing on natural antimicrobial compounds are included. These natural antimicrobial compounds can be used directly, as well as either in a modified or mixed way, so as to increase their activity. You will find, for example, mixtures of chitosan oligomers with ε-polylysine acting as antifungals against three *Botryosphareiaceae* species [16]. Likewise, in the last paper [17], some berberine derivatives can be more potent than berberine itself in attenuating MexXY efflux-dependent aminoglycoside resistance in *Pseudomonas aeruginosa*, demonstrating that natural compounds are not only useful when used in direct applications, but also to obtain derivative compounds with enhanced antimicrobial properties.

The huge amount of natural antimicrobial compound applications and their direct inhibition of bacteria are detailed in terms of a range of applications—for instance, in the avoidance of biofilms. The food industry may well benefit from this in-depth study, and it is likewise useful as a basis from which to obtain more potent compounds. We do expect that this group of manuscripts will be of great help to every scientist or professional interested in the biocontrol of bacteria, fungi or even other biological agents, using a natural alternative instead of the classical chemical compounds.
